# Dystrophin Distribution and Expression in Human and Experimental Temporal Lobe Epilepsy

**DOI:** 10.3389/fncel.2016.00174

**Published:** 2016-07-08

**Authors:** Ruben G. F. Hendriksen, Sandra Schipper, Govert Hoogland, Olaf E. M. G. Schijns, Jim T. A. Dings, Marlien W. Aalbers, Johan S. H. Vles

**Affiliations:** ^1^Department of Neurology, Maastricht University Medical CentreMaastricht, Netherlands; ^2^School for Mental Health and Neuroscience, Maastricht UniversityMaastricht, Netherlands; ^3^Department of Neurosurgery, Maastricht University Medical CentreMaastricht, Netherlands; ^4^Department of Neurosurgery, Groningen University Medical CentreGroningen, Netherlands

**Keywords:** Duchenne muscular dystrophy, dystrophin, epilepsy, seizures, kindling

## Abstract

**Objective:** Dystrophin is part of a protein complex that connects the cytoskeleton to the extracellular matrix. In addition to its role in muscle tissue, it functions as an anchoring protein within the central nervous system such as in hippocampus and cerebellum. Its presence in the latter regions is illustrated by the cognitive problems seen in Duchenne Muscular Dystrophy (DMD). Since epilepsy is also supposed to constitute a comorbidity of DMD, it is hypothesized that dystrophin plays a role in neuronal excitability. Here, we aimed to study brain dystrophin distribution and expression in both, human and experimental temporal lobe epilepsy (TLE).

**Method:** Regional and cellular dystrophin distribution was evaluated in both human and rat hippocampi and in rat cerebellar tissue by immunofluorescent colocalization with neuronal (NeuN and calbindin) and glial (GFAP) markers. In addition, hippocampal dystrophin levels were estimated by Western blot analysis in biopsies from TLE patients, post-mortem controls, amygdala kindled (AK)-, and control rats.

**Results:** Dystrophin was expressed in all hippocampal pyramidal subfields and in the molecular-, Purkinje-, and granular cell layer of the cerebellum. In these regions it colocalized with GFAP, suggesting expression in astrocytes such as Bergmann glia (BG) and velate protoplasmic astrocytes. In rat hippocampus and cerebellum there were neither differences in dystrophin positive cell types, nor in the regional dystrophin distribution between AK and control animals. Quantitatively, hippocampal full-length dystrophin (Dp427) levels were about 60% higher in human TLE patients than in post-mortem controls (*p* < 0.05), whereas the level of the shorter Dp71 isoform did not differ. In contrast, AK animals showed similar dystrophin levels as controls.

**Conclusion:** Dystrophin is ubiquitously expressed by astrocytes in the human and rat hippocampus and in the rat cerebellum. Hippocampal full-length dystrophin (Dp427) levels are upregulated in human TLE, but not in AK rats, possibly indicating a compensatory mechanism in the chronic epileptic human brain.

## Introduction

Dystrophin is an essential component of a protein complex that connects the cytoskeleton to the extracellular matrix. Mutations in the dystrophin gene result in DMD, which is the second most common genetically inherited disease affecting approximately 1 in 3,500-5,000 life male births (Anderson et al., 2002; Emery, 2002; Emery et al., 2015). The absence of dystrophin leads to myofiber membrane fragility that results in the progressive muscular degeneration that characterizes DMD (Sussman, 2002).

Additionally, there is an increasing body of evidence stating that DMD is associated with non-progressive cognitive and behavioral deficits (Cotton et al., 2001; Hendriksen and Vles, 2006, 2008; Pane et al., 2012; Snow et al., 2013a) and epilepsy (Goodwin et al., 1997; Etemadifar and Molaei, 2004; Pane et al., 2013). This association is thought to result from the lack of dystrophin within the CNS, where it is normally located in the hippocampus, prefrontal cortex, and cerebellum (Chamberlain et al., 1989; Lidov et al., 1993). Different CNS cells express different Dp isoforms: full-length dystrophin (Dp427) is localized post-synaptically in neurons, Dp140 is associated with microvascular glia cells, and Dp71 - the most abundant dystrophin gene product in the brain – is both expressed in neurons and glia (Lidov et al., 1995; Blake and Kröger, 2002; Graciotti et al., 2008).

Functionally, dystrophin expressed in the CNS plays an important role in the clustering of neurotransmitter receptors and water- and ion channels to the cellular membrane. The above mentioned three brain dystrophin isoforms have their own distinctive functions: neuronal Dp427 is important for the clustering of GABA_A_ receptors at the post-synaptic membrane and hence for regulation of inhibitory input (Brünig et al., 2002). Similarly, Dp71 is mainly involved in the anchoring and regulation of both the glial water channel AQP-4 and the glial potassium Kir4.1 channel (Connors et al., 2004; Perronnet and Vaillend, 2010; Anderson et al., 2012), thereby regulating water homeostasis and potassium buffering capacity, respectively, (Hendriksen et al., 2015). As water and ion homeostasis have powerful effects on neuronal excitability, alterations in the expression of these dystrophin isoforms may contribute to hyperexcitability associated with epilepsy by the above mentioned disturbances (Hendriksen et al., 2015). Finally, the function of Dp140 is far less well-known: it is expressed in both brain- and kidney tissue, is particularly expressed during fetal development (Lidov et al., 1995) and supposed to play some role in cognitive functioning (Bardoni et al., 2000; Waite et al., 2012). Consequently, its potential theoretical relation with epileptogenesis is hard to speculate upon and hence this isoform will not be further discussed in this paper.

In this study, we evaluated dystrophin expression in human and experimental TLE. Analogous to the preferred method of detection for muscular dystrophin, we used a combination of descriptive immunofluorescence and Western blot (Anthony et al., 2014) to assess dystrophin expression in the hippocampus and cerebellum – two of the major brain structures containing dystrophin -, as the hippocampus plays a major role in the pathogenesis of TLE (Racine et al., 1972; Sloviter, 1994), whereas the cerebellum is supposed to play a potential role in effective anti-epileptic neuromodulation (Velasco et al., 2008; Al-Otaibi et al., 2011). Consequently, the aim of this study was, first, to evaluate regional and cellular dystrophin distribution in the aforementioned two anatomical brain regions by means of immunofluorescence and, second, to assess whether there were quantitative differences in dystrophin expression between epileptic and control individuals by means of Western blot analysis.

## Materials and Methods

### Ethical Statement

All procedures performed in studies involving human participants were in accordance with the ethical standards of the institutional and/or national research committee and with the 1964 Helsinki declaration and its later amendments or comparable ethical standards. Written informed consent was obtained from all individual participants included in the study. All applicable international, national, and/or institutional guidelines for the care and use of animals were followed. All procedures performed in studies involving animals were in accordance with the ethical standards of the institution or practice at which the studies were conducted.

### Amygdala Kindling Model

#### Animals

Male 10-week-old Sprague-Dawley rats, purchased from Harlan (Horst, Netherlands) were kept under controlled conditions (21 ± 2°C ambient temperature, a 12 h light/dark schedule, background noise provided by radio and food and water available *ad libitum*). The animals were habituated 1 week to the experimenter and the housing before the start of the experiment. All experimental procedures were approved by the local animal ethics committee of Maastricht University and complied with national and international governmental legislation.

#### Surgery

Twenty-four rats were implanted with an electrode for AK. Thirty minutes before surgery, the rats received 0.1 ml buprenorphine (Temgesic) subcutaneously for perioperative pain relief. The surgical procedures took place under general isoflurane anesthesia (5% for induction and 2,5% for maintenance). A standard rat stereotact (Stoelting Europe, Dublin, Ireland) was used for implanting the electrode in the rats. The bipolar platinum/iridium needle of the electrode, with a tip diameter of 200 μm, was implanted in the left basolateral amygdala (coordinates relative to bregma: 2,5 mm posteriorly, 4,8 mm laterally, and 9,6 mm ventrally) (Paxinos and Watson, 1986). Also one stainless screw was implanted over the nasal sinus, which served as reference. The connector was fixed on the skull using dental acrylic cement.

#### Amygdala Kindling Procedure

Amygdala kindling started 10 days after the electrode implantation and was performed as described previously (Aalbers et al., 2009, 2014; Rijkers et al., 2010). Initially, stimulation was performed twice daily (first stimulus between 8 and 10 AM, second stimulus between 2 and 4 PM; an interstimulus-interval > 6 h) with the following parameters: 2 s, 400 μA, 50 Hz, 0.2 ms block pulses. A stimulus intensity of 400 μA was chosen to assure that the intensity was above the after discharge threshold for all rats. Stimuli were delivered through a WPI Accupulser A310 connected to a WPI Stimulus Isolation Unit A360 (World Precision Instruments, Sarasota, FL, USA).

All rats were videotaped (Olympus FE-330) during delivery of the kindling stimulus and for as long as the behavioral seizure lasted. Seizure severity was evaluated oﬄine from video-recordings by two blinded observers and classified according to the Racine scale (Racine et al., 1972). After reaching the fully kindled state, defined as five consecutive stage five seizures, rats received one AK-stimulation per day for two more weeks. Sham rats (*N* = 8) received an amygdala electrode that was not stimulated. Rat tissue processing protocols, i.e., for immunofluorescence and Western blot, are further described in the respective sections.

### Human Tissue

#### Surgery

Human hippocampi were intra-operatively obtained between 2010 and 2015 at Maastricht University Medical Centre (MUMC+). All patients suffered from medically refractory TLE (Kwan et al., 2010). Prior to the study, written informed consent was obtained from each patient. Extensive pre-surgical evaluation included video-EEG monitoring, neuropsychological testing and MR-imaging in all patients and occasionally FDG-PET. Surgical hippocampal specimens were evaluated and sclerosis was graded by a trained observer of the pathology department of MUMC+, after which the samples were encoded. In total, 15 TLE patients (mean age ± SEM = 39.7 ± 4.1 years; 8 males) were included, of which nine patients suffered from severe HS, whereas 6 did not show any signs of HS. Hippocampal tissue obtained at autopsy (*N* = 9; mean age ± SEM = 67.3 ± 3.4 years; all male) served as non-epileptic control tissue. Patient characteristics for all included patients are provided in **Table 1**.

**Table 1:** Clinical characteristics of subjects used for Western blotting analysis.

Control	Gender	Age (years)	Cause of death	Pathology	Hippocampus Side	Classification
1	Male	64	Natural	Unremarkable	Right	Non-epileptic
2	Male	66	Natural	Unremarkable	Right	Non-epileptic
3	Male	76	Sepsis, POB	Unremarkable	Left	Non-epileptic
4	Male	80	Heart Failure	Unremarkable	Right	Non-epileptic
5	Male	66	Unknown	Unremarkable	Right	Non-epileptic
6	Male	61	MI-resuscitation	Unremarkable	Right	Non-epileptic
7	Male	83	MI-resuscitation	Unremarkable	Right	Non-epileptic
8	Male	55	Sepsis, MOF	Unremarkable	Unknown	Non-epileptic
9	Male	55	Cardiac after PE	Unremarkable	Right	Non-epileptic

**Case**	**Gender**	**Age (years)**		**Pathology**	**Hippocampus side**	**Classification**

1	Male	35	-	Mesial temporal sclerosis	Right	Epileptic: severely sclerotic
2	Female	50	-	mesial temporal sclerosis	Right	Epileptic: severely sclerotic
3	Male	63	-	Mesial temporal sclerosis	Right	Epileptic: severely sclerotic
4	Female	43	-	Mesial temporal sclerosis and microdysgenesis (possibly in relation to cortical dysplasia)	Right	Epileptic: severely sclerotic
5	Male	19	-	Mesial temporal sclerosis & FCD 2A	Right	Epileptic: severely sclerotic
6	Female	58	-	Mesial temporal sclerosis	Right	Epileptic: severely sclerotic
7	Male	33	-	Mesial temporal sclerosis	Left	Epileptic: severely sclerotic
8	Female	20	-	Mesial temporal sclerosis	Left	Epileptic: severely sclerotic
9	Female	37	-	Mesial temporal sclerosis	Left	Epileptic: severely sclerotic
10	Male	55	-	DNET, right temporal side (WHO dgr. 1)	Right	Epileptic: non-sclerotic
11	Male	16	-	Venous malformations in amygdala	Right	Epileptic: non-sclerotic
12	Female	64	-	Brain aneurysms (communicans posterior and cerebri media)	Right	Epileptic: non-sclerotic
13	Male	41	-	Reactive cortical gliosis	Right	Epileptic: non-sclerotic
14	Male	24	-	History of right-sided skull-base meningeoma	Right	Epileptic: non-sclerotic
15	Female	38	-	Unremarkable	Right	Epileptic: non-sclerotic

*M, Male; F, Female; POB, Post-operative bleeding; MI, Myocardial Infarction; MOF, Multi- Organ Failure; PE, pulmonary embolism; FCD, Focal Cortical Dysplasia; DNET, Dysembryoplastic Neuroepithelial Tumors.*

#### Tissue Preparation

Immediately after surgical resection, the hippocampi were cooled for 1 min at 4°C 0.9% saline and then dissected into two parts perpendicular to the longitudinal axis. One part was fixed in 4% paraformaldehyde overnight at 4°C, embedded in paraffin, and used for routine histopathological evaluation. The other part was immediately frozen on dry ice and stored at -80°C until further analysis (Aalbers et al., 2014).

### Immunofluorescence

#### Rat Tissue

Two or 24 h after the last seizure, rats were sacrificed and processed for immunofluorescence as described previously. Rats received an overdose of pentobarbital (Nembutal, 0.1 mg/kg body weight) and were then *trans*-cardially perfused with 0.5 M ice cold phosphate buffered saline (PBS) followed by 4% paraformaldehyde in 0.5 M PBS. Brains were dissected and post-fixed in 4% paraformaldehyde/0.5 M PBS for 90 min. Next, brains were cryoprotected by immersion in 20% sucrose/0.5 M PBS for 24 h. Subsequently, the brains were frozen by immersion in -40°C isopentane for 3 min and stored at -80°C until further analysis by immunofluorescence.

For immunofluorescence, 50 μm sagital sections of the right cerebellum (50 μm, free floating) and coronal sections of the left hippocampus (30 μm, free floating) were serially cut using a cryostat. Free-floating sections were successively incubated at room temperature for 30 min in 1 M tris**-**buffered saline (TBS), and subsequently with 0.5% Triton X-100, and 10% normal donkey serum (NDS) for 60 min. Then, sections were incubated overnight at 4°C in TBS containing 0.5% Triton X-100 and 10% NDS - for NeuN (neuronal nuclei) in hippocampus - or PBS - for GFAP (glial fibrillary acidic protein) and calbindin in cerebellum and GFAP in hippocampus -, and rabbit polyclonal anti-dystrophin (Abcam, Ab15277, Cambridge, UK, diluted 1:500), followed by donkey anti-rabbit secondary antibody conjugated to Alexa 488 (Invitrogen, Carlsbad, CA, USA, diluted 1:200). For the cerebellum, sections were incubated overnight with monoclonal mouse anti-calbindin D-28k (SWANT, McAB 300, Marly, Switzerland, diluted 1:25,000) as a marker for PC or with monoclonal mouse anti-GFAP (Sigma-Aldrich, G3893, Saint-Louis, MO, USA, diluted 1:500) as a marker for astrocytes. Hippocampal tissue was also stained with monoclonal mouse anti-GFAP (Sigma-Aldrich, G3893, Saint-Louis, MO, USA, diluted 1:500) and additionally with monoclonal mouse NeuN (Millipore, mab377, Darmstadt, Germany, diluted 1:50) as a marker for neuronal nuclei. Primary antibodies were detected by donkey anti-mouse secondary antibody conjugated with Alexa 594 (Invitrogen, Carlsbad, CA, USA, diluted 1:200). Finally, a Hoechst (Sigma-Aldrich, Saint-Louis, MO, USA) staining (1:500) was performed at room temperature and after washing, the sections were coverslipped with 80% glycerol. Negative controls were incubated with non-immune serum. Rat biceps femoris muscle sections were used as a positive control for dystrophin immunofluorescence.

Additionally we performed triple staining - due to the unclear subcellular localisation of the dystrophin protein in the cerebellar sections - with the following primary anti-bodies: rabbit anti-dystrophin (Abcam, Ab15277, Cambridge, UK, diluted 1:500), monoclonal mouse anti-calbindin D-28k (SWANT, McAB 300, Marly, Switzerland, diluted 1:2,500) and polyclonal goat anti- GFAP (Santa Cruz Biotechnology, C-19, Heidelberg, Germany, diluted 1:200). All primary antibodies were diluted in 1% NDS in 0.5% PBS-T and simultaneously incubated at 4°C for 16 h after blocking for 1 h with 10% NDS. Secondary antibodies used were: Donkey anti-mouse secondary antibody conjugated to Alexa 647 (Abcam, Cambridge, UK, diluted 1:200), donkey anti-rabbit secondary antibody conjugated to Alexa 488 (Abcam, Cambridge, UK, diluted 1:200), donkey anti-goat secondary antibody conjugated to Alexa 594 (Invitrogen, Carlsbad, CA, USA, diluted 1:100). All secondary antibodies were diluted in 1% NDS in 0.5% PBS-T and simultaneously incubated at room temperature for 2 h. After washing, the sections were coverslipped with 80% glycerol.

#### Human Hippocampus Tissue

Paraffin-embedded human hippocampal sections of 4 μm were cut in a coronal plane and directly mounted on glass. Sections were deparaffinized, followed by antigen-retrieval (0.01 M citric acid in a water bath of 95°C for 20 min, followed by 20 min of cooling). Sections were immunohistochemically stained as described above using the following antibody concentrations: rabbit anti-dystrophin (1:100), mouse anti-GFAP (1:500), NeuN (1:50), Alexa 488 (1:100), and Alexa 594 (1:100).

#### Image Acquisition

Photomicrographic images were made using a BX51-microscope (Olympus, Tokyo, Japan) connected to an Olympus XC10 camera (Olympus, Tokyo, Japan), which generated 16-bits TIFF files. Images were taken with different exposure times for the different fluorophores. Exposure times were kept constant among all sections, thereby only being filter-specific (DAPI/Hoechst 1 ms, TXRED 1000 ms, and FICT 1000 ms). Additionally, confocal 1 μm space stacks were made of neuronal and glial cells in hippocampal sections (600 times magnification), PC in cerebellar sections (1000 times magnification, not shown) and the cerebellar triple staining (400 times magnification) by means of a confocal disk spinning unit microscope (Olympus, Tokyo, Japan) financed by The Netherlands Organisation for Scientific Research (grant number 911-06-003). In the immunostainings of human tissue, all pictures were taken from the GCL of the DG.

### Western Blot

#### Tissue Preparation

Rats were sacrificed 2 (i.e., acute group, *N* = 8) or 24 h (i.e., chronic group, *N* = 8) after their last seizure. For the collection of hippocampal samples, rats were decapitated, brains were removed, right-side hippocampi were isolated by dissection and frozen at -80°C until further analysis. The right side was chosen since this is contralateral to the stimulation site, hence preventing potential confounding by the effects of direct electrical stimulation.

Each hippocampus (i.e., rat and human) was separately homogenized in lysis buffer (1 g tissue per 9 ml lysis buffer) containing 0.01 M PBS, 1% Igepal, 0.1% Triton X-100, 1 mM EGTA (ethylene glycol tetraacetic acid), 1 mM EDTA (ethylenediaminetetraacetic acid), and protease inhibitor (Roche Custom Biotech, ML758, Penzberg, Germany).

For the limited amount of rat cerebellar tissue available (sham control and AK acute group; both *N* = 4), proteins were extracted from paraformaldehyde fixed sections using an extraction buffer kit according to the manufacturer’s instructions (Qiagen, Venlo, the Netherlands).

#### Western Blot Protocol and Quantification

After estimating the total protein concentration per sample using a bovine serum albumine standard curve (BioRad, Hercules, CA, USA), proteins (40 μg per lane for homogenate) were separated by gel electrophoresis using a 7.5% polyacrylamide gel containing 20% SDS. Each sample was run in duplicate. Proteins were subsequently transferred to a PVDF membrane (Merck Millipore, Billerica, MA, USA) for 16 h at 35V. After a 2 h blocking step in commercial Odyssey blocking buffer (diluted 1:2 with PBS and 5% NDS), the membrane was incubated overnight at 4°C with polyclonal rabbit anti- dystrophin (Abcam, Ab15277, Cambridge, UK, diluted 1:100) and subsequently for 1 h with goat anti-rabbit IRDye 800CW (LI-COR, Homburg, Germany, diluted 1:10,000) in blocking buffer. Rat muscle (m. biceps femoris) was used as a positive control and only showed the Dp427 isoform (not shown). Immunoreactive protein bands were visualized by an Odyssey infrared imaging system (Li-COR Biosciences, Lincoln, NE, USA) (Cooper et al., 2003). Optical density values were normalized to glyceraldehyde-3-phosphate dehydrogenase (GAPDH), detected by mouse monoclonal anti-GAPDH primary (Fitzgerald Industries International, Acton, MA, USA, diluted 1:2,000,000) and donkey anti-mouse IRDye 680RD (LI-COR, Homburg, Germany, diluted 1:10,000) secondary antibody.

Immunoblots were analyzed with ImageJ software (Anthony et al., 2014). Relative pixel intensities were measured and the background signal was subtracted (Taylor et al., 2013). Every dystrophin isoform was corrected for by its respective GAPDH expression and subsequently expressed as a percentage of the average of the sham’s rats or post-mortem control patients’ expression on the respective gel (Anthony et al., 2014). During quantification, the observer was blinded to the experimental condition to prevent biased assessment. Typical examples of western blots are depicted in **Figures 6C,F**, and **7C**.

#### Data Analysis

Quantified blot intensities were averaged of at least two independent experiments (and thus two or more samples per animal) within groups and were expressed as the mean + SEM. These values were then used to calculate group averages. In order to evaluate the effect of kindling on dystrophin expression, we compared levels of sham and kindled animals by a Mann-Whitney *U* test. To compare the acute effect with the long-lasting effect of a seizure on dystrophin expression in the hippocampus, we compared rats that were sacrificed 2 and 24 h after a seizure by, again, a Mann-Whitney *U* test. Because two tests were performed, we corrected the alpha of 0.05 by Bonferroni to 0.025.

Similarly, for human hippocampi, we compared dystrophin levels in epileptic and non-epileptic tissue, using a Mann-Whitney *U* test. In order to assess the influence of neuronal cell loss and gliosis, we also evaluated the difference in dystrophin expression between epileptic patients with and without HS, according to an alpha level of 0.025.

## Results

### Regional and Cellular Brain Dystrophin Distribution

#### Rat Brain Dystrophin Distribution

**FIGURE 1:**
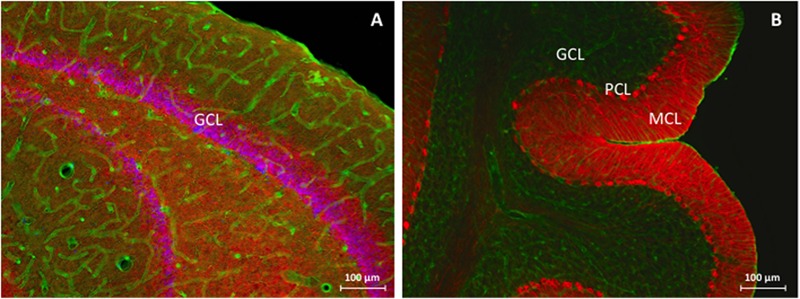
**Immunofluorescent overview staining of rat hippocampus **(A)** and rat cerebellum **(B)** in order to evaluate dystrophin expression in an animal model for TLE, 100 times magnification. (A)** Hippocampal DG, consisting of the GCL is stained purple due to colocalization between NeuN (red) and Hoechst (blue) and is surrounded by dystrophin (green) positive astrocytes and blood vessels through all different hippocampal layers. **(B)** The three cerebellar layers can be clearly distinguished: from right to left note the completely red MCL as a consequence of densely packed PC processes, the PCL as visualized by the calbindin (red) antibody, and finally the GCL which mainly consists of dystrophin (green) positive cells and blood vessels. GCL, granular cell layer, MCL, molecular cell layer, PCL, Purkinje cell layer.

Dystrophin distribution was studied in rat hippocampus and rat cerebellum. A general overview of these examined anatomical regions is depicted in **Figure 1** by means of *low-power images.*

##### Hippocampus

**FIGURE 2:**
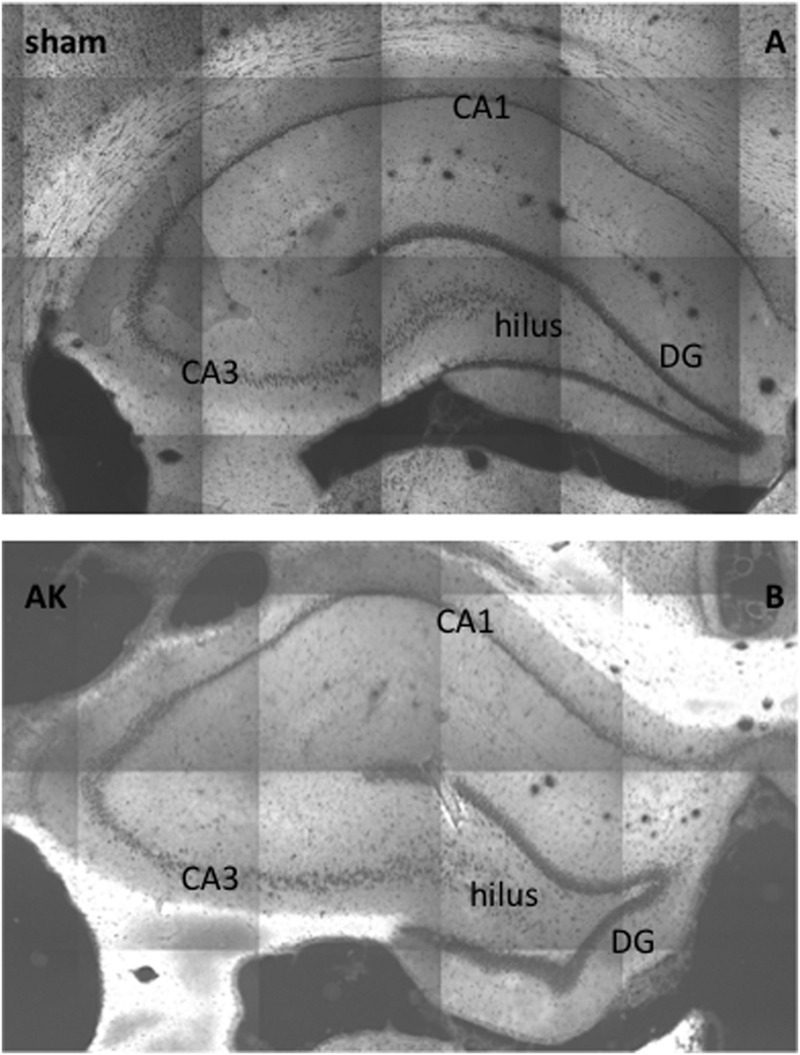
**H&E-staining of (dorsal) hippocampus of a sham rat **(A)** and AK rat **(B)**, coronal section, 20 times magnification.** No global differences in morphology (e.g., granular cell dispersion or pyramidal cell loss) could be noted. CA = cornu ammonis; DG = dentate gyrus.

**FIGURE 3:**
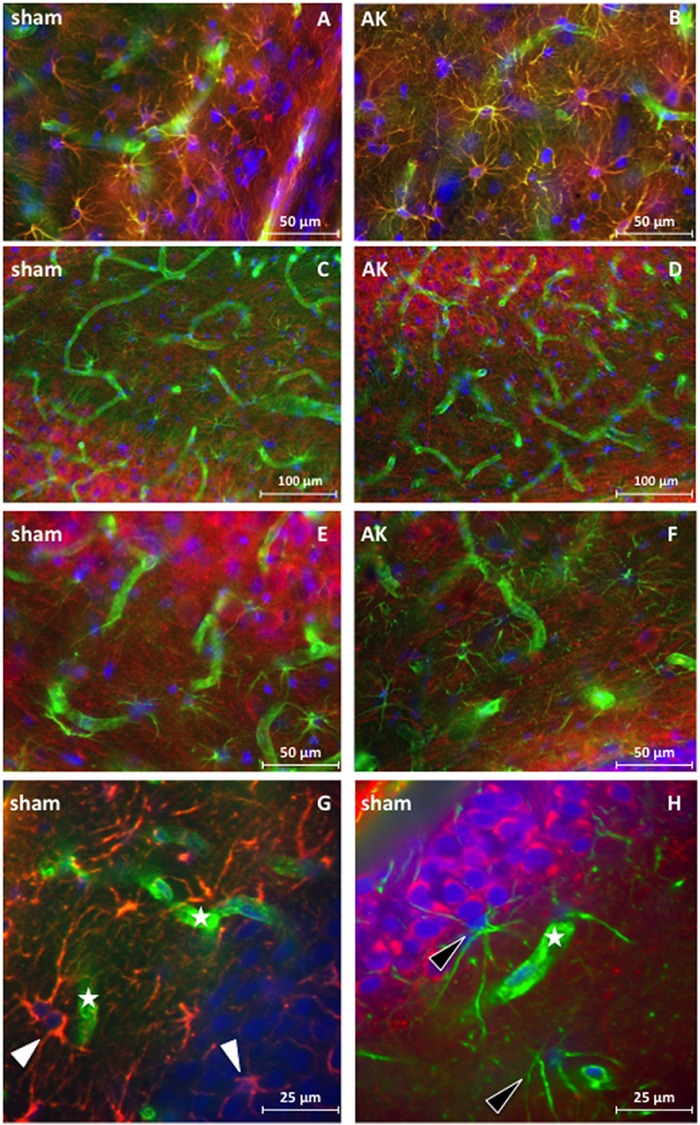
**Immunofluorescent staining of dystrophin distribution in rat hippocampus.** Double staining for dystrophin (green) and GFAP (red) (magnification 400 times) reveals colocalization (yellow signal) of GFAP and dystrophin in rat hippocampal sections of a control rat **(A)** and a similar colocalization pattern of GFAP and dystrophin is present in the hippocampus of AK rats (acute condition) **(B)**. No colocalization is present in the double-labeled immunofluorescent staining for dystrophin (green) and NeuN (red) (magnification 200 times) in and around the pyramidal cell layer of the CA3 region in sham rat tissue **(C)** nor in AK (acute) rat **(D)**. There is also no colocalization between dystrophin (green) and NeuN (red) (magnification 400 times) in the hilus between the DG and CA3 in control rat hippocampus **(E)** or AK (acute) rat hippocampus **(F)**. **(G,H)** (sub)cellular distribution of dystrophin in rat hippocampus (DG) as visualized by confocal microscopy (magnification 600 times). **(G)** One micrometer spacing stack through the hippocampus of a sham rat stained for dystrophin (green) and GFAP (red), white arrowheads indicate dystrophin positive, colocalizing astrocytes. **(H)** One micrometer spacing stack through the hippocampus of a sham rat stained for dystrophin (green) and NeuN (red), black arrowheads indicate dystrophin positive (perivascular) astrocyte-like cells. Asterisks indicate dystrophin positive blood vessels. Sham = control rat, AK = Amygdala Kindled rat (here: acute AK rat; i.e., sacrificed 2 h after the last seizure).

Morphologically, no global changes (e.g., granular cell dispersion) or differences were detected when comparing sham rat hippocampus with AK rat hippocampus as proven by H&E staining (**Figure 2**). Dystrophin is present in multiple layers of the hippocampus. In the rat hippocampus it is ubiquitously expressed around the pyramidal cell layer of CA1 through CA4 as well as in the hilus and around the GCL of the DG. The morphological appearance of dystrophin suggests expression of dystrophin in blood vessels and astrocytes (**Figures 3A-H**). Indeed there is a strong colocalization between dystrophin and GFAP positive astroglia (**Figures 3A,B,G**). Both in the pyramidal cell layer and in the GCL, there was no colocalization present between dystrophin and the neuronal marker NeuN (**Figures 3C-F,H**). The regional and cellular distribution of dystrophin did not differ between sham control and AK rats.

##### Cerebellum

**FIGURE 4:**
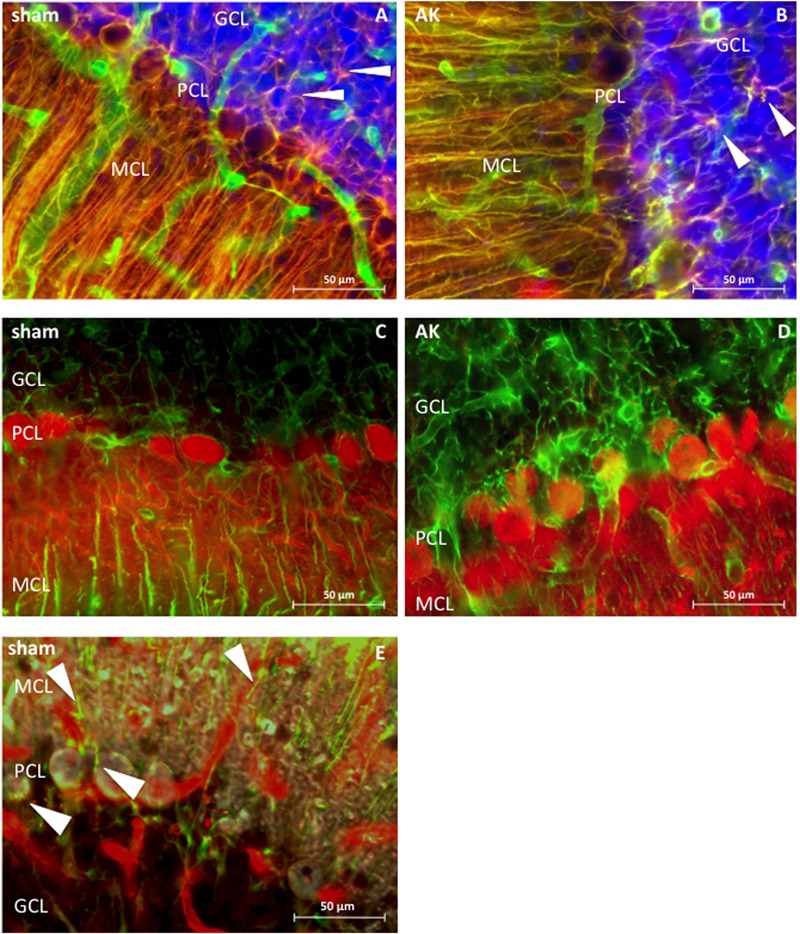
**Dystrophin distribution in rat cerebellum (sagital sections), 400 times magnified. (A)** Sham rat section stained for dystrophin (green) and GFAP (red). **(B)** AK (acute) rat section stained for dystrophin and GFAP. Note the colocalization between GFAP and dystrophin (in both **A,B**), hence revealing BG in yellow, which are extending from the molecular layer and surround the PC. There is also co-expression in the GCL between GFAP and dystrophin, thereby possibly reflecting dystrophin positive velate protoplasmic astrocytes (white arrowheads). **(C)** Dystrophin (green) and calbindin (red) expression in sham rat cerebellum. **(D)** Dystrophin and calbindin expression in the cerebellum of an (acute) AK rat. There is no clear co-expression of dystrophin with calbindin positive PC in **(C,D)**. **(E)** Triple staining in sham/control rat tissue with dystrophin (red), GFAP (green), and calbindin (gray) visualized by means of confocal microscopy, solely performed in order to assess which cell types here express dystrophin. Dystrophin is visible in blood vessels and in BG (indicated by white arrowheads), both around the PC - where the soma of the BG is located - but also in the processes within the MCL. Calbindin is depicted in gray and stains the PC as such, yet does not colocalize with dystrophin. Note the presence of multiple blood vessels in all three layers of all images. Sham = control animal, AK = Amygdala Kindled animal (here: acute AK rat; i.e., sacrificed 2 h after the last seizure).

Dystrophin is present in the MCL, the PCL, and GCL of the cerebellum (**Figure 4**). Morphologically, it appears to be present around PC, in astrocytes of both the MCL and GCL, and in blood vessels across all three cerebellar layers. The somata of PC located within the PCL are surrounded by dystrophin immunoreactivity, hence either reflecting PCs or BG. Triple-immunostaining (**Figure 4E**) shows clear colocalization with GFAP but not with the neuronal calbindin protein, thus confirming the dystrophin positive BG surrounding individual PC. The diffuse dystrophin immunoreactivity in the GCL and the colocalization with GFAP reflects dystrophin positive velate protoplasmic astrocytes in this layer (**Figures 4A,B**). The regional and cellular distribution of dystrophin did not differ between sham control and AK rats.

#### Human Brain Dystrophin Distribution

**FIGURE 5:**
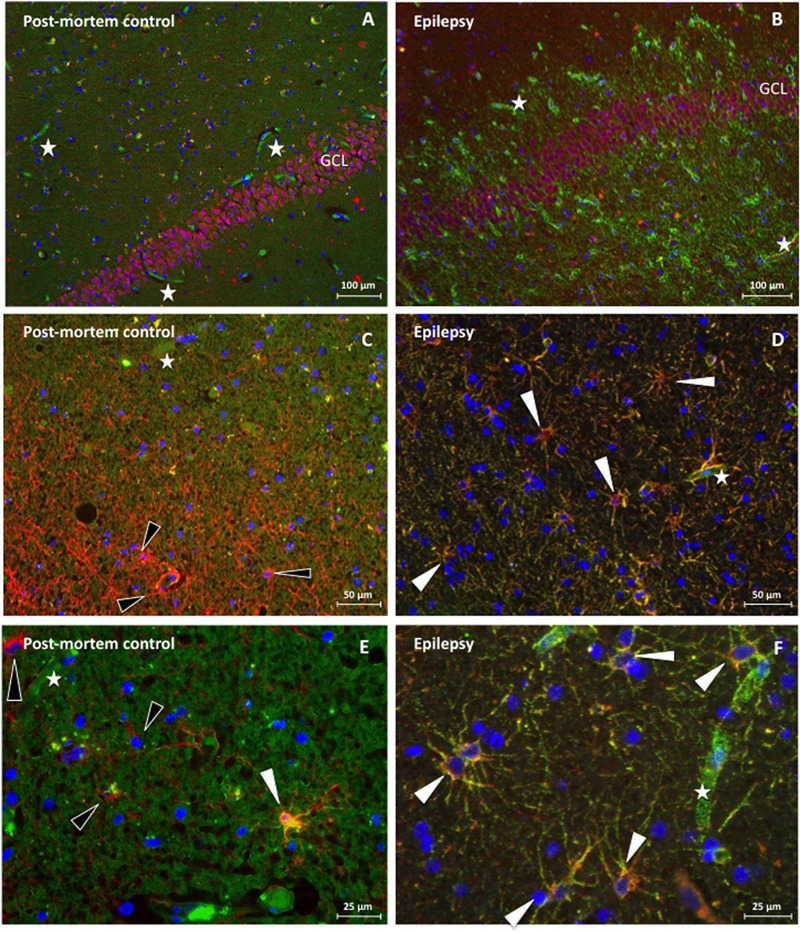
**Immunofluorescence of dystrophin distribution in human hippocampus of a post-mortem control patient **(A,C,E)** and a TLE patient without sclerosis **(B,D,F)**. (A)** NeuN (red) in the GCL of the DG without dystrophin (green) positive astroglial-like cells in post mortem control tissue. Next, none of these NeuN positive cells are stained yellow and are therefore dystrophin negative, 100 times magnification. **(B)** NeuN staining revealing NeuN (red) in the GCL of the DG and many dystrophin (green) positive astroglial-like cells in the hippocampus (DG) of a TLE patient, 100 times magnification. Again, no colocalization is observed. **(C)** GFAP (red) but not dystrophin (green) positive astrocytes (indicated by means of black arrowheads) around the DG in the hippocampus of a post-mortem control patient, 200 times magnification. **(D)** Dystrophin positive astrocytes around the DG of an epileptic hippocampus, revealing clear (yellow) colocalization, double stained for dystrophin (green) and GFAP (red), 200 times magnification. **(E)** 400 times magnified close-up of the DG of a post-mortem control patient. The white arrowhead indicates a glial cell that reveals some colocalization between GFAP (red) and dystrophin (green), whilst the black arrowheads indicate none colocalizing astrocytes. **(F)** 400 times magnified close-up of an area within the DG of a TLE patient. The white arrowheads depicted indicate, again, glial cells that show (yellow) colocalization between GFAP (red) and dystrophin (green). Asterisks indicate dystrophin positive blood vessels.

Analogous to the rat hippocampal tissue, the morphological appearance of dystrophin in human hippocampal tissue suggests expression of dystrophin in blood vessels and astrocytes (**Figure 5B**). Indeed strong colocalization between dystrophin and GFAP positive astroglia appears again (**Figures 5D,F**). Based on a general visual examination in all performed stainings, the amount of dystrophin positive astrocytes seems to be higher in the epilepsy patient (**Figure 5D**) (here: non-sclerotic) compared to the post-mortem control (**Figure 5C**). That is, here, only one astrocyte is stained yellow in this post-mortem control tissue, whereas three others (black arrowheads) are red (**Figure 5E**), compared to at least six yellow astrocytes and zero red, none-colocalizing astrocytes, in the non-sclerotic epileptic tissue of **Figure 5F**. In the NeuN/dystrophin double-staining no colocalization pattern was found in post-mortem control tissue (**Figure 5A**), nor in TLE patients within the epileptic conditions (**Figure 5B**). It should be noted that the dystrophin distribution pattern was similar for sclerotic and non-sclerotic patients, therefore no visual distinction could be made and hence only the images of an epileptic, non-sclerotic patient are shown here.

### Quantitative Brain Dystrophin levels in Epilepsy

#### Amygdala Kindled Rats

**FIGURE 6:**
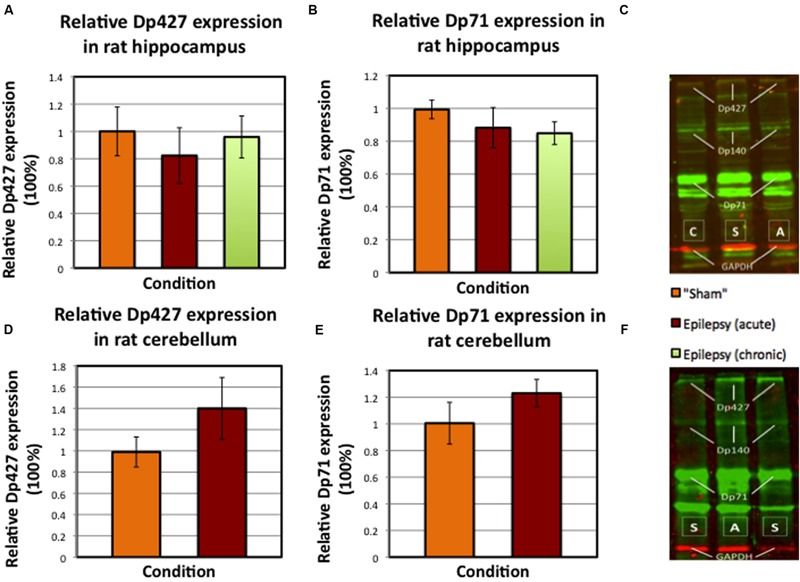
**Mean values of relative dystrophin expression in hippocampus and cerebellum of sham and AK rats (SEM’s are indicated with whiskers). (A)** Dp427 expression in sham rats (*N* = 8) versus acute AK (*N* = 7) and chronic AK rats (*N* = 8). **(B)** Dp71 expression in again sham rats (*N* = 8) versus acute AK (*N* = 8) and chronic AK (*N* = 8) rats. **(C)** An example of the performed Western blot technique for dystrophin (in green) in rat hippocampus. The different isoform bands as well as the GAPDH bands (red) are indicated. **(D)** Dp427 expression in the cerebellum of sham rats (*N* = 4) versus (acute) AK rats (*N* = 4). **(E)** Dp71 expression in again sham rats (*N* = 4) and (acute) AK rats (*N* = 4). **(F)** Typical example of Western blot performed in cerebellum. S = sham/control rat, A = acute AK rat (sacrificed 2 h after last seizure), C = chronic AK rat (sacrificed 24 h after last seizure).

**FIGURE 7:**
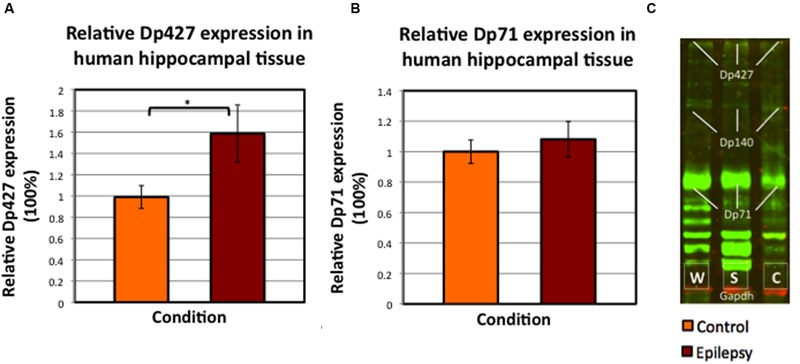
**Mean values of relative dystrophin expression in the hippocampus of patients operated for therapy-resistant TLE by means of neurosurgical removal of the hippocampus, compared to post-mortem controls (SEM’s are indicated with whiskers). (A)** Dp427 expression in post-mortem controls (*N* = 8) versus epilepsy patients, both with severe sclerosis and without sclerosis (total *N* = 15). ^∗^Significant at alpha < 0.025. **(B)** Dp71 expression in again hippocampus from post-mortem control patients (*N* = 9) and in hippocampus from all patients included with TLE (i.e., with and without severe sclerosis; *N* = 15). **(C)** Example of Western blot performed in human hippocampus tissue; it is interesting to note the aspecific dystrophin (green) bands, especially below Dp71. Abbreviations in panel C: W = without/no sclerosis, S = severe sclerosis, C = post-mortem control.

Western blotting demonstrated that the full-length isoform (Dp427), Dp140, and Dp71 are expressed in the hippocampus and cerebellum of AK rats (see **Figures 6C,F and 7C** for a typical example).

The relative dystrophin expression for both isoforms (i.e., Dp427 and Dp71) in rat hippocampus was on average neither statistically significantly different between sham and AK rats (**Figures 6A,B**, sham vs. all AK rats pooled *p* = 0.506 for Dp427 and *p* = 0.081 for Dp71), nor between acute AK and chronic AK rats (**Figures 6A,B**, acute AK vs. chronic AK *p* = 0.694 for Dp427 and *p* = 0.88 for Dp71). In the cerebellum no statistical differences could be detected either (**Figures 6D,E**, *p* = 0.343 for Dp427 and *p* = 0.686 for Dp71).

#### Human TLE

For the control tissue, the post-mortem delay time was minimized (average time ± SEM = 1112 ± 175 min). In addition, dystrophin expression intensity did not correlate with post-mortem delay (Pearson’s *R* = 0.28, *p* = 0.47); hence the post-mortem delay did not influence the analysis.

Dp427 expression was on average 59.8% higher (SEM post-mortem control group: 10.7%; SEM pooled epilepsy group: 26.9%) in TLE patients (*N* = 15; *p* = 0.023), whereas Dp71 expression was not significantly increased in epileptic patients (+8.4%; *p* = 0.682; **Figure 7**). Furthermore, there was no statistically significant difference in dystrophin expression between severely sclerotic and non-sclerotic hippocampi from TLE patients (data not shown, *p*-values of 0.689 and 0.955 for Dp427 and Dp71, respectively).

## Discussion

Here, we evaluated the hippocampal and cerebellar distribution of dystrophin in TLE patients and in an animal model of TLE (I) qualitatively by means of immunofluorescence in order to assess both the regional and cellular distribution pattern, and (II) quantitatively by means of Western blot analysis. We have shown that dystrophin is expressed in different types of glial cells in multiple layers of both the hippocampus and the cerebellum. Surprisingly, no expression of dystrophin in neuronal cells – like granular cells in the hippocampus or PC in the cerebellum – was found. In rats, the dystrophin distribution pattern did not seem to differ between sham control and AK conditions. Furthermore, the amount of dystrophin expression was not different between control and kindled animals. In human hippocampi from TLE patients there was, however, a significant increase in the amount of Dp427 when pooling all TLE samples.

### Regional and (Sub) Cellular Brain Dystrophin Distribution

It is unclear why we did not detect neuronal dystrophin expression in rat tissue, as the used dystrophin antibody is directed against the carboxy-terminal of the protein and hence supposed to detect all isoforms as also demonstrated by Western Blot. Possibly, the abundantly present Dp71, which is the most numerous dystrophin isoform in the brain (Lederfein et al., 1992; Aleman et al., 2001), may have overshadowed the expression of Dp427 as it may have led to saturation of the antibody. Yet increasing antibody concentrations as well as augmented incubation times did not alter the expression pattern. Moreover, Dp71 is also present in neurons (Austin et al., 2000; Aleman et al., 2001) and nonetheless no colocalization was observed.

The presence of dystrophin in cerebellar PC has been previously debated, i.e., whereas some studies report on expression in the soma (including cytoplasm and plasma membrane) and the dendritic tree (Huard and Tremblay, 1992), others show that dystrophin is confined to the somatic membrane (Snow et al., 2013b). The diffuse cytoplasmic labeling might be explained by the use of biotinylated antibodies in some studies (Huard and Tremblay, 1992), which may bind to endogenous biotin in PC (Snow et al., 2013b) or by the presence of auto-fluorescence, which is known to occur in the center of PC somata through the accumulation of lipofuscin (Barden, 1980; Schnell et al., 1999). In this study, we checked for auto-fluorescence by microscopic evaluation of unstained sections, which confirmed its absence.

The immunoreactivity surrounding the soma of the PC here might be attributable to Dp71 (Moukhles and Carbonetto, 2001; **Figure 4**) and thus might reflect BG as their cell bodies are located in the PCL. Besides, these specific glial cells are known to intimately embrace PC (Tanaka et al., 2008). Indeed, a triple staining performed in this study showed colocalization of dystrophin with GFAP but not calbindin (**Figure 4E**). In the rat cerebellum dystrophin was immunohistochemically also present in astrocytes within the GCL, thereby possibly representing velate protoplasmic astrocytes (Hoogland and Kuhn, 2010), which is in contrast to the finding that dystrophin immunoreactivity in the GCL is limited to blood vessels (Blake et al., 1999). Additionally, dystrophin was present in astrocytes in the MCL (**Figure 4**), thereby reflecting Dp71 positive BG as proposed earlier (Moukhles and Carbonetto, 2001).

### Quantitative Dystrophin Levels in Epileptic Brain Tissue

Based on quantitative Western blot analysis, there were no significant differences in dystrophin expression between kindled and sham control animals. In line with our results, hippocampal dystrophin levels were not altered in other pre-clinical epilepsy models such as the pentylenetetrazole and status epilepticus model (Gorecki et al., 1998; Kim et al., 2010). Knuesel et al. (2001), on the other hand reported regional specific alterations by making use of the KA model for TLE, a model that is followed by progressive morphological alterations (Bouilleret et al., 1999). This model demonstrated a Dp71 upregulation in the hippocampal molecular layer, yet a downregulation in the GCL (Knuesel et al., 2001). The contradicting effect of up- and downregulation within the same structure might have led to a net difference of approximately zero, which may explain the results in our study in which a homogenate of the full hippocampus was evaluated. However, we could not detect any major regional differences by visual inspection of the immunofluorescent stained rat hippocampus sections.

In contrast, the human Western blot results demonstrated increased Dp427 levels in hippocampi from drug-resistant TLE patients compared to post-mortem controls. This finding is in contrast to the studies hitherto performed that revealed a reduction of hippocampal dystrophin (Lee et al., 2004; Das et al., 2012) or even an absence of perivascular Dp71 from sclerotic hippocampus, specifically in the CA1 region (Eid et al., 2005). However, such Dp71 alterations might be region specific since in the subiculum of the same HS patient a strong dystrophin signal could be observed in and around the capillaries, which was furthermore comparable to the signal observed in the non-HS subiculum (Eid et al., 2005). In contrast, here we did not observe such substantial differences in any sclerotic hippocampal region (images not shown). Yet, as can be deduced from these recordings, dystrophin is visible in and around the GCL and MCL of the DG in severely sclerotic hippocampus. Furthermore, the Western blots (**Figure 7C**) in this study instantly reveal expression of Dp71 in the hippocampus of epilepsy patients with severe sclerosis, which may thereby further support the notion of region specific alterations in hippocampal dystrophin, as earlier described by Knuesel et al. (2001) in rat tissue.

The dystrophin increase (here: only Dp427) in epileptic hippocampi could theoretically be explained as a compensatory upregulatory mechanism. Dp427 and Dp71 have powerful effects on excitation by clustering of postsynaptic GABA_A_-receptors (Brünig et al., 2002) and astrocytic AQP-4 and Kir4.1 channels (Connors et al., 2004; Perronnet and Vaillend, 2010), respectively, (Hendriksen et al., 2015).

However, non-neuronal dystrophin, as shown in this study by means of immunohistochemical stainings, does not only cluster the aforementioned two channels but also plays a role in blood-brain barrier functioning. That is, Dp71 binds actin filaments between endothelial cells and is consequently able to influence vascular permeability (Tinsley et al., 1994). This way, Dp71 may, again, be related to hyperexcitation (Hendriksen et al., 2015). Additionally, evidence suggests that Dp71 clusters post-synaptic proteins that, in turn, organize glutamatergic transmission (Daoud et al., 2009), thereby illustrating another relation with (hyper) excitation and consequently TLE.

Based on the previous, an upregulation of the DMD gene could result in more dystrophin transcription and hence multiple potential compensatory mechanisms that could restore the balance between excitation and inhibition in brains that are prone to hyperexcitation. This could also partly clarify why the prevalence of epilepsy is increased in patients with DMD. Notwithstanding the fact that a dystrophin absence could theoretically also result in epilepsy (Pane et al., 2013; Hendriksen et al., 2015), a mutation in the DMD gene also impairs the occurrence of the abovementioned potential compensatory mechanism. Furthermore, the lack of such mechanisms could theoretically be, analogous to the cognitive deficits that are often seen in DMD (Ricotti et al., 2016), mutation dependent; a more distal mutation affects more brain isoforms and would thus give rise to multiple deficient counteracting mechanisms (i.e., both neuronal and glial mediated), hence increasing the probability of hyperexcitable brain (networks) and thereby epilepsy.

The discrepancy between human and experimental TLE data regarding dystrophin expression is interesting. Possibly, this can be explained by the presence of neuronal cell loss and gliosis in human TLE, which is absent in AK since this model is not characterized by neurodegeneration (Brandt et al., 2004). Indeed, Heuser et al. (2012) demonstrated a difference in dystrophin immunoreactivity in epilepsy patients with and without hippocampal sclerosis. However, in this study we did not find such a difference in dystrophin expression between these two groups. Another possible explanation is that in humans, the tissue from which the seizure originated was evaluated, while in rats the contralateral hippocampus was used in order to exclude the influence of direct electrical stimulation. Moreover, in contrast to the kindled rats, all human patients suffered from chronic and intractable epilepsy that has been tried to manage for years before epilepsy surgery was performed. The compensatory mechanisms hypothesized above may only develop over years *in-vivo*, and not after the relatively short period (i.e., 3 weeks) of experimental seizures.

### Limitations and Future Perspectives

This study also has its limitations. First of all, the full-length isoform was based on immunofluorescence neither visualized in cerebellum nor in hippocampus. Additionally, for ethical reasons we were not able to include human cerebellar tissue in this study. For rats, cerebellar rat tissue was treated differently in the Western blot procedure since it was paraformaldehyde fixed. As the material from the experimental AK group has been treated in the same way, this should not have affected the relative difference between the two groups. Furthermore, the two groups included in the analysis of the cerebellum are small. Inevitably, the average age of the post-mortem control patients was – logically – substantially higher than the average age of the epilepsy patients, which could have influenced the quantitative expression of neuronal proteins.

Future research should focus on studying dystrophin expression in different brain areas in order to better understand the possible association between hyperexcitation and dystrophin alterations, since this relation has only been marginally addressed in literature. Conversely the role of dystrophin in (hyper)excitation should be further evaluated using animal models in which different dystrophin isoforms are absent, ideally by making use of electrical kindling techniques, such as in this study applied and which has not been studied before. Ultimately, future studies should disentangle the possible contribution of the different dystrophin isoforms to epileptogenesis in order to assess possibilities for new anti-epileptic therapies.

## Conclusion

Dystrophin is ubiquitously expressed in the hippocampus and cerebellum of AK rats and in human hippocampal tissue from epilepsy patients with and without HS as proven by Western blot and immunofluorescent analysis in this study. Dp427, which principal function in the brain it is to anchor GABA_A_ receptors in the post-synaptic membrane, is increased in human epileptic hippocampi, possibly as a compensatory mechanism in order to restore the inhibitory balance in hyperexcitable brains. This is the first study that reports on a (full-length) dystrophin upregulation in epilepsy, which justifies more research in order to further investigate this newly emerging relationship between hyperexcitation and dystrophin. Future studies should address how the different brain dystrophin isoforms are related to hyperexcitation, in what direction this relationship may be established and, ultimately, whether dystrophin may represent a novel target for seizure treatment.

## Author Contributions

All authors agree to be accountable for the content of the work. RH and SS performed the experiments. RH and MA performed the statistical analysis. MA obtained the animal tissue used in this study. OS and JD obtained the human tissue used in this study. MA and GH supervised the laboratory experiments. RH, MA, and JV drafted the manuscript, after which all authors substantially contributed to obtain the final version. JV initiated this research project and furthermore supervised and guided the collective research process.

## Conflict of Interest Statement

The authors declare that the research was conducted in the absence of any commercial or financial relationships that could be construed as a potential conflict of interest.

## Abbreviations

AK: amygdala-kindling
AQP-4: aquaporin 4
BG: Bergmann glia
CA: cornu ammonis
CNS: central nervous system
DG: dentate gyrus
DMD: Duchenne muscular dystrophy
Dp: dystrophin protein
EEG: electroencephalography
FDG-PET: fluorodeoxyglucose positron emission tomography
GABA_A_: gamma-aminobutyric acid type A
GCL: granular cell layer
GFAP: glial fibrillary acidic protein
HS: hippocampal sclerosis
KA: kainic Acid
Kir4.1: inwardly rectifying potassium
MCL: molecular cell layer
MR: magnetic resonance
NeuN: neuronal nuclei
PC: Purkinje cell
PCL: Purkinje cell layer
TLE: temporal lobe epilepsy
